# Anthocyanin-Dyed Cotton Enhanced with Lavender Oil Microcapsules: A Dual Approach for Color Stability and Sustained Fragrance Release

**DOI:** 10.1021/acsomega.4c09486

**Published:** 2025-05-30

**Authors:** Rafael Grande, Kelcilene B. R. Teodoro, Isabela S. Bertho, Isabela F. Pinheiro, Alessandra R. P. Ambrozin, Daniel S. Correa, Débora T. Balogh, Rafaela C. Sanfelice

**Affiliations:** † Grande Apresentações, Rua Paraná, 229, Centro, Poços de Caldas, Minas Gerais 37701-018, Brazil; ‡ Nanotechnology National Laboratory for Agriculture (LNNA), 564899 Embrapa Instrumentation, Rua XV de Novembro, 1452, Centro, São Carlos, Sao Paulo 13561-206, Brazil; § Science and Technology Institute, 74347 Federal University of Alfenas (ICT/UNIFAL), Rodovia José Aurélio Vilela, 11999, BR 267 Km 533, Poços de Caldas, Minas Gerais 37715-400, Brazil; ∥ São Carlos Institute of Physics, 67817 University of São Paulo, Av. Trabalhador São-carlense, 400, Parque Arnold Schimidt, São Carlos, Sao Paulo 13566-590, Brazil

## Abstract

This study explores the development of cotton fabrics with enhanced color durability and controlled fragrance release through the microencapsulation of lavender oil using chitosan and carboxymethyl cellulose (CMC) in combination with anthocyanin-based dyeing. The innovation of this work lies in the application of sustainable biopolymers to improve both the aesthetic and functional properties of textiles, addressing the growing demand for eco-friendly solutions in the industry. The encapsulation process was designed to prolong the release of lavender oil, enhancing its functional properties for therapeutic textile applications. Microcapsules were prepared by emulsifying lavender oil in a solution containing anthocyanin and chitosan followed by their incorporation into a CMC solution to yield stable capsules. The structural integrity of the microcapsules was analyzed by optical microscopy, and their interaction was confirmed via FTIR analysis, which revealed strong hydrogen bonding and electrostatic interactions between chitosan and CMC. The controlled release of lavender oil was evaluated with the microcapsule-impregnated fabric showing a slower release rate (diffusion constant of 1.53 × 10^–3^ min^–1^) compared to direct oil impregnation. Color stability tests demonstrated the resilience of anthocyanin-dyed fabrics to light exposure, with minimal photodegradation observed after 5 days under continuous illumination. These results highlight the potential of biopolymers such as chitosan and CMC in dyeing and encapsulation processes, offering sustainable methods for the development of multifunctional and eco-friendly textiles.

## Graphical abstract

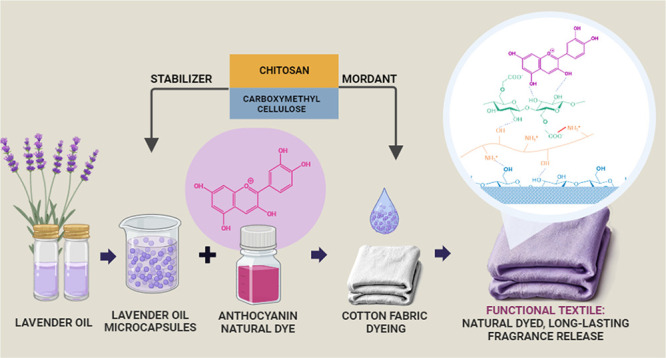

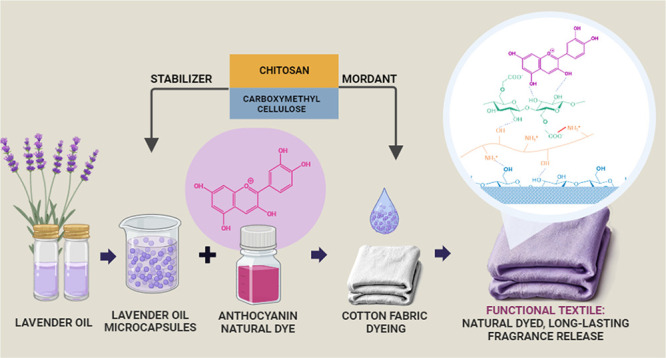

## 1 Introduction

The textile industry is shifting toward sustainability due to environmental concerns and increasing demand for eco-friendly solutions.^1^ Synthetic dyes and harsh chemicals traditionally used in dyeing processes harm both the environment and human health, necessitating alternatives that combine functionality with reduced ecological impact.^2^ As a result, there is a significant push within the industry to develop alternative dyeing methods that minimize ecological impact while enhancing fabric functionality.^3^ One promising approach involves the use of natural dyes (such as anthocyanins) and biopolymers [such as chitosan and carboxymethyl cellulose (CMC)], the combination of which could offer not only aesthetic benefits but also additional properties for the textiles, such as biodegradability, antimicrobial activity, and UV protection.

Anthocyanins are a class of flavonoid compounds responsible for the red, purple, and blue colors found in many fruits, vegetables, and flowers. Chemically, anthocyanins are glycosides of anthocyanidins, i.e., anthocyanidin (aglycone) linked to one or more sugar units.^4,5^ The most common anthocyanidins include cyanidin, delphinidin, malvidin, pelargonidin, peonidin, and petunidin.^6^ The color of anthocyanins is influenced by the number and position of hydroxyl and methoxy groups on the anthocyanidin structure, as well as by medium pH.^7^ In acidic conditions, anthocyanins typically appear red, while in neutral to alkaline conditions, they shift to purple or blue hues. These pigments are extracted from various plant sources such as berries (blueberries, blackberries, raspberries), grapes, red cabbage, and eggplants, where they play significant roles in plant propagation and defense mechanisms.^5,8^ The extraction process typically involves the use of polar solvents, such as methanol or ethanol, often acidified to stabilize the flavylium cation form of anthocyanins, which is responsible for their vibrant colors. The stability and functionality of these compounds make them suitable for various applications in both food science and pharmacology.^5,6,9^

Chitosan is a natural biopolymer obtained by the deacetylation of chitin found in the shells of crustaceans. It is recognized for its antimicrobial properties, biocompatibility, and biodegradability,^10–12^ making it an excellent material for the encapsulation of bioactive compounds. Chemically, chitosan is composed of repeating units of β-(1→4)-linked d-glucosamine and *N*-acetyl-d-glucosamine, giving it unique physicochemical properties such as solubility in acidic solutions and the ability to form films, hydrogels, and nanoparticles.^9,13,14^ The presence of amino groups allows chitosan to interact with various molecules through hydrogen bonding and ionic interactions, making it highly versatile for encapsulation purposes.^14–16^

CMC is a water-soluble cellulose derivative produced through the reaction of cellulose with chloroacetic acid. It is widely used in several industries due to its unique properties, such as high viscosity, solubility in cold and hot water, and ability to form films and gels.^17^ CMC contains carboxymethyl groups (−CH_2_–COOH) bound to some of the hydroxyl groups of the glucopyranose monomers that make up the cellulose backbone, imparting a negative charge to the polymer in aqueous solutions. This anionic nature allows CMC to interact electrostatically with positively charged molecules, making it an effective stabilizer, thickener, and emulsifier.^18^ In textile applications, CMC is used to enhance the binding of dyes and pigments to fibers, improve the uniformity of dye uptake, and provide additional functionality such as increased fabric strength and durability.^3^ The combination of CMC with other biopolymers, such as chitosan, improves the complementary properties of these materials, resulting in advanced systems with enhanced performance and environmental benefits.^19^

The combination of chitosan and CMC enhances the anchoring and dyeing of cotton fabrics with anthocyanins by exploiting the strong interactions between their polymeric chains. Chitosan’s protonated amino and hydroxyl groups interact electrostatically with CMC’s carboxylate and hydroxyl groups, forming a stable dual-layer matrix. This matrix facilitates the attachment of anthocyanins, improving color retention in the fabric.^20,21^ This method not only ensures attachment of anthocyanins, enhancing color fastness and durability, but also protects the pigments from degradation, maintaining their vibrant hues.^9^ Moreover, the use of these biopolymers offers an environmentally friendly alternative to synthetic dyes, aligning with the growing demand for sustainable textile processing methods.^4,8,22^

The selection of chitosan and CMC was based on their chemical and functional complementarity. The choice of the encapsulation technique via polyelectrolyte complexation was guided by its efficiency and simplicity, eliminating the need for organic solvents or thermal processing steps that could degrade bioactive compounds. Unlike methods such as spray-drying or thermal coacervation, the complexation between chitosan and CMC occurs under mild conditions, preserving the essential oil’s properties while ensuring the formation of stable capsules on the fabric surface. These characteristics make the adopted approach a sustainable and functional solution for the development of multifunctional textiles, integrating natural dyes with prolonged fragrance release in a single system. In addition to their eco-friendly nature, chitosan and CMC were specifically chosen over other polyelectrolyte pairs due to their biocompatibility, biodegradability, and tunable interactions. Alternative polyelectrolyte pairs such as alginate-gelatin or pectin-chitosan were considered but presented limitations.^23,24^

The application of chitosan and CMC in textile dyeing with anthocyanins exemplifies the potential of biopolymer-based systems in advancing eco-friendly technologies. This innovative approach not only improves the aesthetic qualities of the fabric but also imparts additional functionalities such as antimicrobial properties and UV protection, making it suitable for various industrial applications.^9^

Lavender essential oil is a valuable natural product that is used in traditional medicine, cosmetics, and aromatherapy. Commercial lavender oil, primarily derived from Lavandula angustifolia, L. spica, and L. hybrida, is valued for its calming and soothing properties due to its main components, linalool, linalyl acetate, and camphor.^25–27^ These volatile compounds are present in the essential oil in the range of 25–38, 25–45, and 0.5–1.0%, respectively, and are known to influence the central nervous system, reduce anxiety, and have mild sedative effects.^28,29^ Usually, higher amounts of camphor in the ratio of those three main compounds decrease the quality and the economic value of lavender oil.^26^ Lavender oil is widely used in aromatherapy for its stress-reducing and sleep-enhancing properties. Its ability to penetrate the skin and slowly release aroma properties make it ideal for textiles requiring a continuous fragrance release.^30,31^ In this context, here we propose an innovative approach that combines the aesthetic appeal of anthocyanin-dyed fabrics with the therapeutic benefits of lavender oil, creating textiles that are both visually pleasing while promoting well-being.^28,32,33^

Microcapsules have emerged as an alternative method for integrating functional properties into fabrics. By encapsulation of active substances, such as antimicrobial agents, fragrances, phase-changing materials, or vitamins, within a protective shell, microcapsules can enhance the durability and controlled release of these compounds under specific environmental stimuli such as friction, pH changes, or temperature. For instance, chitosan has been extensively employed as a microencapsulation agent, enabling applications ranging from cosmetic textiles to medical fabrics.^34–36^ Moreover, advanced encapsulation methods, including complex coacervation, spray-drying, and layer-by-layer deposition, have been developed to produce microcapsules with tailored size, morphology, and stability, ensuring effective integration with textile substrates through finishing techniques such as padding, impregnation, and grafting.^36,37^ These innovations have broadened the scope of functional textiles, enabling enhanced thermal regulation, prolonged fragrance release, and improved resistance to environmental degradation, thus addressing the growing demand for high-performance and sustainable textile solutions.^38^

In this context, the microencapsulation of lavender essential oils can benefit from utilizing natural polymers, such as chitosan and CMC. These biopolymers form stable protective shells around the oil, slowing its evaporation and enabling a controlled aromatic release. These biopolymers are advantageous due to their biocompatibility, biodegradability, and ability to form stable microcapsules that protect the encapsulated oil and control its release rate.^39^ The microencapsulation process involves the formation of a protective matrix around the oil, which slows its evaporation and prolongs its aromatic effect. This technique not only enhances the longevity of the fragrance but also allows for targeted and controlled delivery. This study represents an integration of natural dyes and essential oils into textile applications, combining the aesthetic and functional properties of anthocyanin-dyed cotton with the therapeutic benefits of lavender oil microcapsules. By employing biopolymers such as chitosan and CMC for both dye stabilization and microencapsulation, this work not only enhances the longevity and vibrancy of natural colors but also introduces a sustainable approach to controlled fragrance release. The dual functionality achieved through this methodology contributes to meeting the growing demand for eco-friendly and multifunctional textiles, offering potential for applications in therapeutic and advanced fabrics.

Although our approach focuses on sustainable materials, synthetic encapsulation systems, such as those based on polyurethane (PU), have also been widely explored in the literature. For example, Özsevinç and Alkan (2022 and 2023)^40,41^ synthesized PU-based microcapsules for the release of lavender oil using propylene glycol as a more environmentally friendly alternative to traditional diols like ethylene glycol. Their results demonstrated excellent mechanical stability and controlled release properties, with microcapsule sizes ranging from 15 to 50 μm and encapsulation efficiencies of up to 52%. However, despite the enhanced mechanical properties and controlled release performance of PU-based systems, these methods often involve petrochemical-derived components, which raise concerns regarding environmental impact and sustainability. In contrast, our study utilizes chitosan and CMC, natural and biodegradable polymers, which meet the growing demand for environmentally friendly alternatives in textile applications. Although natural systems may exhibit lower mechanical strength compared to synthetic polymers, they offer sufficient stability and controlled release behavior, as demonstrated by the results presented in this work. Our findings suggest that natural biopolymer-based encapsulation systems represent a promising and sustainable alternative for textile applications, especially when the focus is on reducing environmental impact. Future studies could explore ways to enhance the mechanical stability of natural systems while maintaining their biodegradability and performance.

## 2 Experimental Section

### 2.1 Materials

Chitosan with 74% deacetylation and a viscometric molar mass of approximately 33,000 g/mol was employed (Galena Química). Sodium CMC SKU was obtained from Sigma-Aldrich, and anthocyanin was obtained from CHR Hansen (ColorFruit Red 108 WSP GIN 720109). The lavender essential oil was obtained from ViaAroma. The cotton fabric was a white woven 100% cotton denim, 250 g/m^2^, EPI (ends per inch) of 80, and PPI (picks per inch) of 70, typically used for laboratory coats.

### 2.2 Preparation of Cotton Fabric

The fabric was cut into strips (2 × 4 cm—approximately 197 mg) and submitted to a rigorous washing process to remove any impurities. The fabric was washed with neutral soap according to the manufacturer’s instructions. After washing, the fabric was rinsed several times to ensure complete removal of the soap. The fabric was then dried in the shade at room temperature.

### 2.3 Dyeing of Cotton Fabric

The dyeing conditions for the fabric were based on the work of Grande et al.^9^ The dyeing of cotton fabric using anthocyanins was conducted by two different methods. In the first method, the dried cotton fabric strips were immersed in a 0.1% (w/v) chitosan solution prepared in 1% (v/v) acetic acid for 30 min. Next, the fabric was rinsed with water and air-dried. Once dry, the fabric was then immersed in an anthocyanin solution (0.1% w/v in 1% acetic acid) for 30 min. After the dyeing process, the fabric was rinsed with water and dried again. In the second method, the fabric strips were first immersed in 0.1% (w/v) chitosan solution prepared in 1% acetic acid for 30 min. After the chitosan treatment and subsequent rinsing and drying, the fabric was immersed in 1% (w/v) CMC solution for 30 min. Next, the fabric was immersed in the anthocyanin solution (0.1% w/v in 1% acetic acid) for 30 min. After this, the fabric was rinsed with water and dried. For comparison purposes, an additional set of cotton fabric strips were immersed only in the anthocyanin solution (1% acetic acid) for 30 min without any prior treatment with chitosan or CMC. After the dyeing process, these strips were also rinsed with water and dried.

To evaluate the interaction between the components of the fabric dyeing, different numbers of bilayers or trilayers were obtained (1–4), repeating the process the required number of times.

### 2.4 Fabrication of Microcapsules Containing Chitosan/CMC/Lavender Oil

To prolong the release time of lavender oil from the fabric, microcapsules were formed through the polyelectrolyte complexation between chitosan (cationic) and CMC (anionic).^42^ This interaction promotes water expulsion and forms an insoluble film, encapsulating the lavender oil and providing structural integrity to the microcapsules.^20,43^ Chitosan exhibits considerable solubility in slightly acidic pH; therefore, all solutions—chitosan, CMC, and anthocyanin—were prepared using 1% acetic acid solution to maintain the pH between 4 and 5, preventing chitosan precipitation. Initially, 200 μL of Tween 80 and 2.5 mL of lavender oil were added to 20 mL of 0.1% (w/w) anthocyanin solution in water, with anthocyanin serving as a color indicator for observing the capsules (Figure 1). The solution was stirred until the oil was completely dissolved. Subsequently, 10 mL of 0.5% chitosan solution was incorporated, and the mixture was stirred until homogeneous. Separately, a CMC solution with the same concentration was prepared and vigorously stirred by using a magnetic stirrer. The chitosan, anthocyanin, and lavender solution were then added dropwise (10 mL/h) to the CMC solution under continuous stirring to form microcapsules. After mixing, the suspension was stirred vigorously for an additional 30 min to ensure homogeneity and capsule formation.

**Figure 1:**
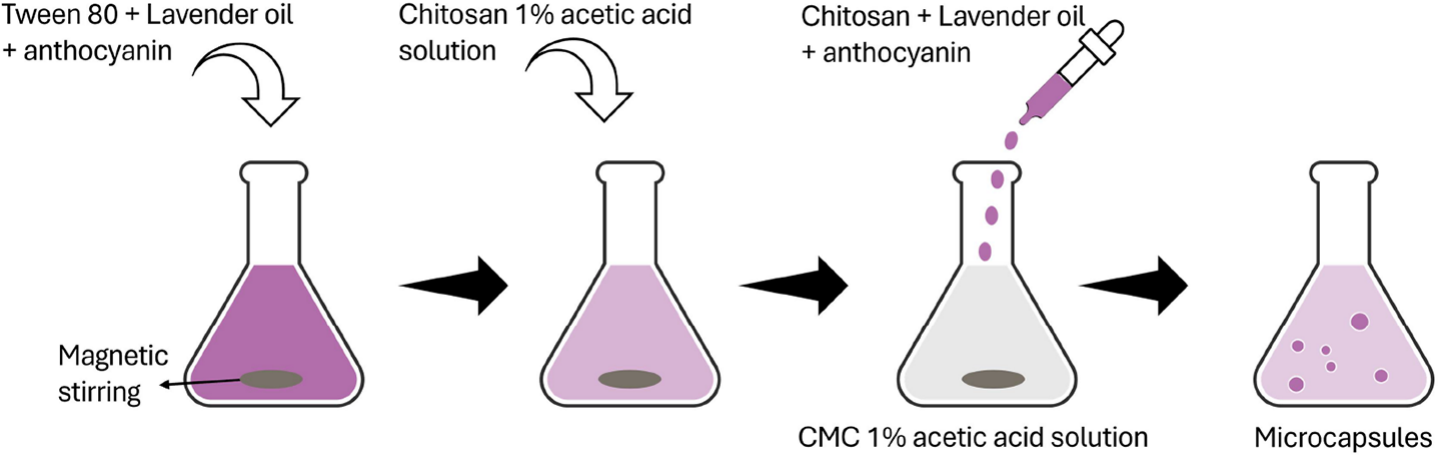
Illustrative scheme of the synthesis of chitosan and CMC microcapsules containing lavender oil and anthocyanin.

### 2.5 Controlled Release Study of Lavender Oil

Lavender oil was diluted in ethanol PA in a ratio of 10% (v/v), and an aliquot was applied to the desired fabric area. The fabric was then left to dry in the shade at room temperature based on the methodology described by Khanna et al.^44^ The same procedure was used for impregnating the microcapsules in the fabric. To evaluate the controlled release of lavender oil, the dry fabric containing chitosan, CMC, and anthocyanin was weighed before applying the lavender solution. After the ethanol fully evaporated, the fabric’s mass was recorded at defined intervals. To corroborate the results, both the lavender solution in ethanol and the microcapsules were deposited on a quartz slide, and the release was evaluated by measuring the variation in UV–vis absorption over time by using a Hitachi U-2001 spectrophotometer in the transmission mode.

### 2.6 Characterizations

The interactions between the molecules composing the microcapsules, namely, chitosan, CMC, and anthocyanins, were investigated using Fourier transform infrared (FTIR) spectroscopy. The measurements were carried out on a ThermoNicolet Nexus 470 instrument in the transmittance mode. The spectra were recorded in the range of 500–4000 cm^–1^, with a total of 64 scans at a resolution of 4 cm^–1^.

The UV–vis absorption spectra of anthocyanins and lavender solutions were evaluated using a Hitachi U-2001 spectrophotometer in the transmission mode, providing insights into the light absorption properties of the solutions.

The characterization of material’s surface morphology was performed using a JEOL JSM 6510 scanning electron microscope. For this, the samples were prepared on glass substrates as a cast film and coated with gold using a sputter coater.

Photodegradation experiments were conducted at room temperature under illumination using a 50 W, 12 V, Osram white halogen light source with an intensity of 17 mW/cm^2^, positioned 30 cm away from the samples. This setup was used to study the stability of the samples under light exposure.

The UV–vis spectra of the anthocyanin-dyed fabrics were recorded using an HR2000 Ocean Optics spectrophotometer, operating from 360 to 1100 nm with a QR600-7-SR125BX two-arm reflectance optical fiber. The absorbance was directly calculated by SpectraSuite software, using a white standard as the reference, through reflectance measurements.

The microcapsules were analyzed by optical microscopy using an Olympus BX41 microscope at magnifications of 40× and 100× in the transmission mode.

## 3 Results and Discussion

### 3.1 Characterization of Cotton Fabric Dyed with Anthocyanin

The UV–vis spectrum of anthocyanin solution exhibits a prominent peak at approximately 520 nm, typical for anthocyanins in an acid solution (pH ∼ 4.0).^45^ This peak indicates the predominant presence of the flavylium form of anthocyanins, responsible for the intense red color of the solution in this pH range, as can be observed in the inset of Figure S1 in the Supporting Information. The presence of a smaller peak around 280 nm can be attributed to the core structure of the anthocyanidins, reflecting the absorption of aromatic groups.^4,8,46^

1% (w/v) anthocyanin solution was used to dye the cotton fabrics and to enhance the fixation on the fabric; two different polymer combinations were tested: neat chitosan and chitosan/CMC. The digital images of the fabrics dyed with anthocyanin (Figure S2 in the Supporting Information), using chitosan/CMC as mordants, clearly show variations in the dye fixation. There is a variation in the intensity and uniformity of coloration among the different samples, suggesting that the interaction between the mordants and the anthocyanins significantly affects the adherence and saturation of the color in the fabric. The intensity of the coloration in the fabrics shows variation, which can be attributed to the degree of interaction between the positively charged chitosan and the negatively charged CMC with the anthocyanins.^19,46,47^ In addition to their charges, the molecules of chitosan, CMC, and anthocyanin contain numerous OH groups, which enable strong interactions (hydrogen bonds), contributing to better color fixation on the fabric.^48^

Figure 2A–D presents the UV–vis spectra of dyed fabric illustrating that, for both mordant treatments—neat chitosan and the chitosan/CMC combination—the absorption intensity increases correspondingly with the number of applied layers. This indicates enhanced dye uptake and fixation with each additional layer. This increase is most notable around 550 nm, which is the characteristic absorption region for anthocyanins,^45,46^ indicating a higher amount of dye retained in the fabrics when more layers are applied. For fabrics with chitosan, a sharp increase in the first few layers is observed, which tends toward equilibrium, as seen in the spectra. For the fabrics where chitosan/CMC were used as mordants, the growth became more pronounced from the second layer onward. The use of chitosan as a mordant, besides the presence of charge that facilitates fixation, is also due to the bacteriological properties of this biopolymer;^49^ however, as the charge of chitosan is positive and that of anthocyanin in acidic solution is also positive, the use of CMC is a good alternative to better fix the dye on the fabric fibers and confer antimicrobial properties to them. The electrostatic interactions and potential cross-linking between anthocyanins and the two polymers create a denser and more stable matrix for the dye. This interaction likely contributes to the improved color intensity and is expected to enhance the durability of the dye under washing and light exposure, which can be further investigated through additional colorfastness tests.

**Figure 2:**
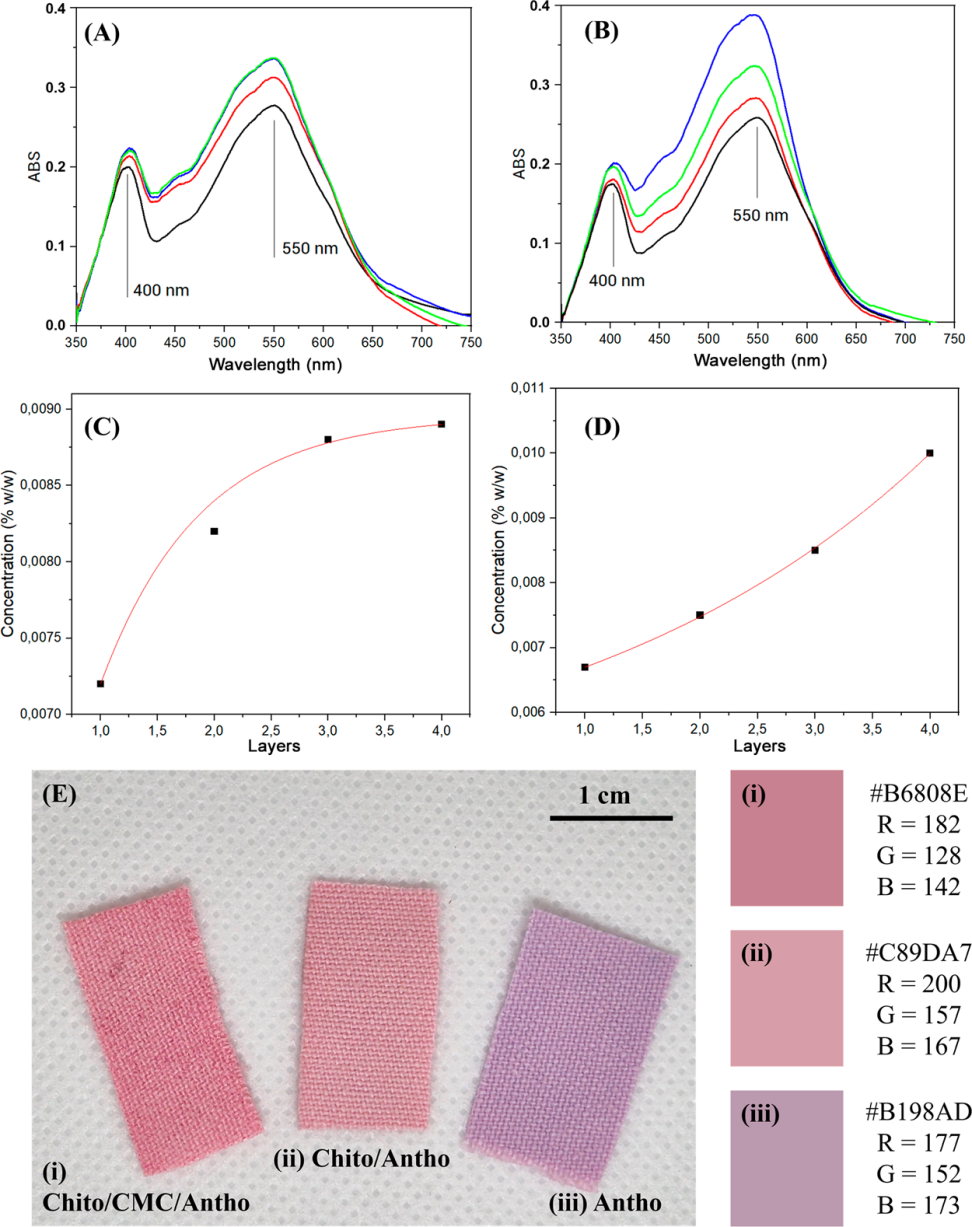
UV–vis spectra of fabrics dyed with anthocyanin: Using (A) chitosan and (B) chitosan/CMC as mordants with 1, 2, 3, and 4 layers. (C, D) Relationship between the anthocyanin concentration and the number of layers. (E) Digital images of fabrics with a single layer of anthocyanin using (i) Chito/CMC as a mordant, (ii) Chito as a mordant, and (iii) without a mordant. Adjacent to the image are the corresponding fabric colors with their respective RGB (red, green, and blue) color proportions.

In Figure 2E, the resulting colors of fabrics dyed with anthocyanin, with and without mordants, are presented. It is evident that the fabric treated with both chitosan and CMC as mordants exhibits a more intense pink coloration compared with the fabric that used only chitosan as a mordant. In contrast, the fabric without any mordant displays a color shift toward violet. The RGB (red, green, and blue) values (Figure 2E i–iii) corroborate these observations, showing a higher proportion of blue in the fabric without a mordant. This color variation in the presence of contaminants highlights the strong interaction between the polymer and dye molecules. Given that the color of anthocyanin is highly dependent on its charge, the presence of charges from chitosan and CMC significantly influences the dye’s color expression. This suggests that the interaction between the charged polymer and the anthocyanin molecules alters the dye’s chromophore environment, leading to the observed color differences.

As a natural dye, anthocyanin is prone to degradation under environmental conditions (upon light exposure, pH changes, etc.), which can impact its stability and color vibrancy. Particularly, excessive light exposure can break down its chemical structure, leading to significant color fading over time.^50^ To evaluate the photodegradation effect, fabrics treated with a single layer of Chito/CMC/Antho were exposed to continuous halogen light source for 5 days (simulating natural lighting), and the results are shown in Figure 3.

**Figure 3:**
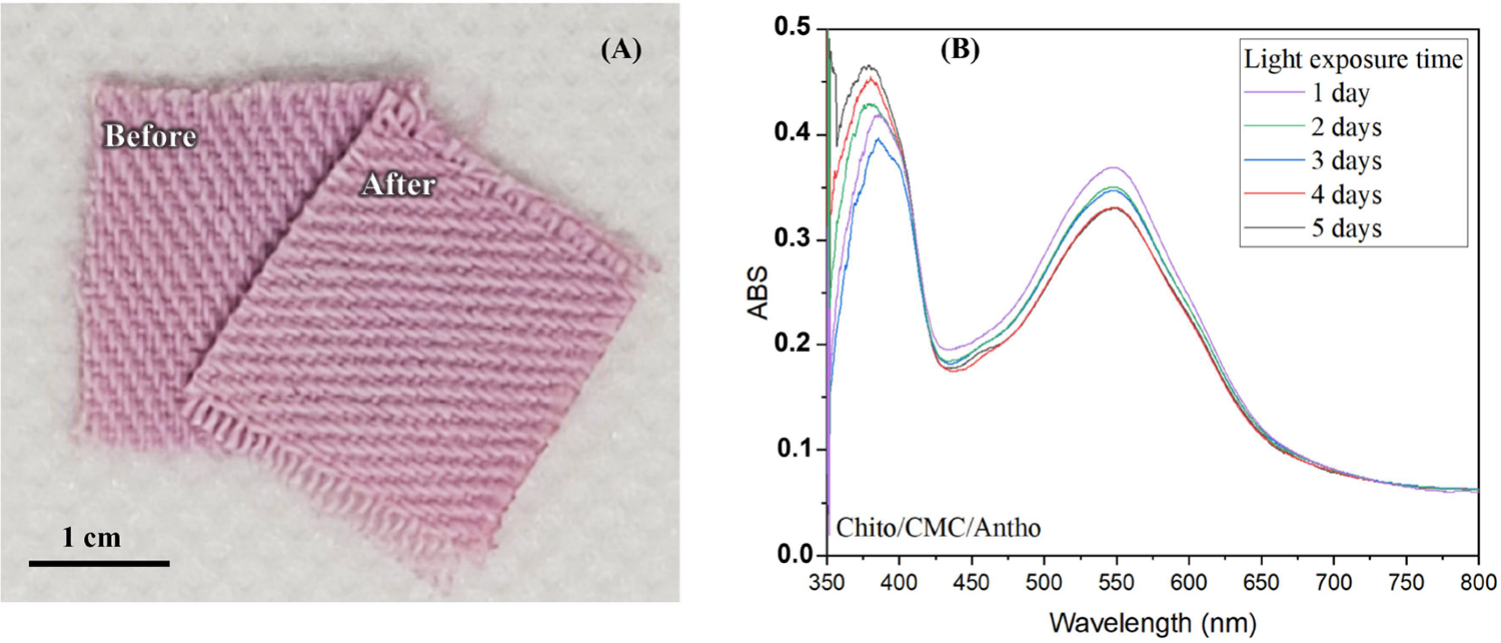
(A) Images of fabrics dyed with anthocyanin and chitosan/CMC as mordants with 1 layer before and after constant light exposure for 5 days. (B) UV–vis spectra of the fabric during this period.

Visual inspection (Figure 3A) and UV–vis spectra (Figure 3B) show minimal fading of dyed fabrics after 5 days of light exposure, indicating the resilience of the anthocyanin dye. Absorbance at ∼520 nm decreased gradually with a more pronounced decline in the initial days. This trend indicates steady photodegradation of the dye molecules under continuous light exposure. The rate of color loss appears to be more pronounced in the initial days, as indicated by the sharper decline between days 1 and 3. The rate then seems to slow down, suggesting that a substantial proportion of the less stable dye molecules might have degraded in the initial phase of exposure.

Anthocyanins degrade primarily due to their susceptibility to photolytic and oxidative processes when exposed to light. The presence of oxygen and light can generate reactive oxygen species, further accelerating the breakdown of the anthocyanin structure into less colored or colorless forms.^50^ While chitosan/CMC is used to enhance the uptake and fixation of the dye on the fabric, its role in protecting against photodegradation is limited. The data suggest that although these polymers might stabilize the dye to some extent, they do not significantly inhibit the photodegradation pathway of anthocyanins.^42,45^

Although anthocyanin-dyed fabrics treated with chitosan and CMC show some stability under light exposure, the gradual decrease in absorbance over time indicates that photodegradation occurs. The slower degradation rate observed after the initial days, however, suggests that the chitosan-CMC-anthocyanin complex may offer a protective effect against further breakdown, consistent with findings on the role of chitosan as a mordant and stabilizing natural dyes.^9^ While this study focused on validating the dual approach concept, these results provide preliminary evidence of potential long-term stability benefits, warranting further investigation under varied conditions such as washing and pH changes.

The color of anthocyanins is highly pH-dependent, shifting from red under acidic conditions to purple and blue as the pH increases. This sensitivity influences color stability, particularly in textile applications, where hue consistency is essential. The structural changes in anthocyanins induced by pH variations alter their chromophore, the part of the molecule responsible for color. As the pH moves toward neutrality or alkalinity, the flavylium cation form of anthocyanins, which is stable in acidic environments, transitions to the quinonoidal base. This transformation not only shifts the color but can also lead to the formation of colorless carbinol pseudobases and chalcones under increasingly alkaline conditions.^45,51^

To further investigate the degradation behavior, the fabric was subjected to pH variation. Initially, the dry fabric was immersed in a slightly basic NaOH solution (pH ∼ 8.0) and then, after washing with water to remove the excess base, it was placed in 1% acetic acid solution. The colors of the fabric after this procedure can be seen in Figure 4A. After five cycles, a marked color loss of the fabric was observed, as shown in Figure 4B.

**Figure 4:**
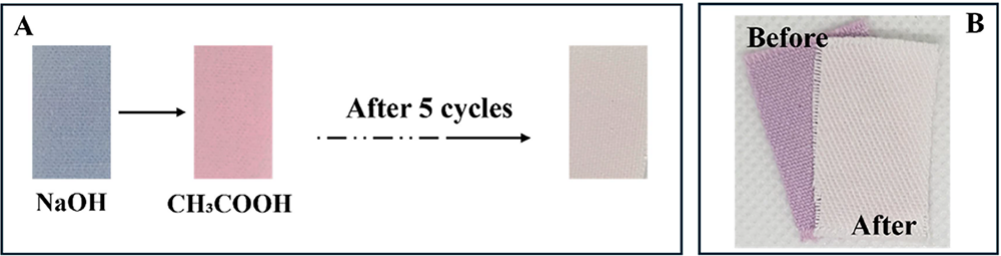
(A) Image of the fabrics after treatment with NaOH and 1% acetic acid (CH_3_COOH) and (B) comparison of the fabric colors before and after five cycles.

Scanning electron microscopy (SEM) was used to observe the morphology of fabric samples dyed with anthocyanin with and without the application of chitosan/CMC mordants. The obtained images (Figure 5) reveal significant differences in the surface structure of the samples, indicating how the application of mordants affects the distribution and fixation of anthocyanins on cotton fibers. SEM images of fabrics dyed without mordants (Figure 5A) reveal smooth fibers with minimal fibrils, indicating limited anthocyanin fixation and resulting in reduced color intensity and durability. On the other hand, when chitosan is used as a mordant (Figure 5B), SEM images show a noticeable change in the surface morphology of the fabric. Many fibrils can be observed throughout the sample, indicating that chitosan effectively adheres to the cotton fibers and creates an additional layer that retains the anthocyanins. This denser coverage suggests better dye fixation, as corroborated by UV–vis data that show higher anthocyanin absorption. The presence of this chitosan layer not only increases the color intensity but can also confer additional properties, such as bacteriological resistance.^9^

**Figure 5:**
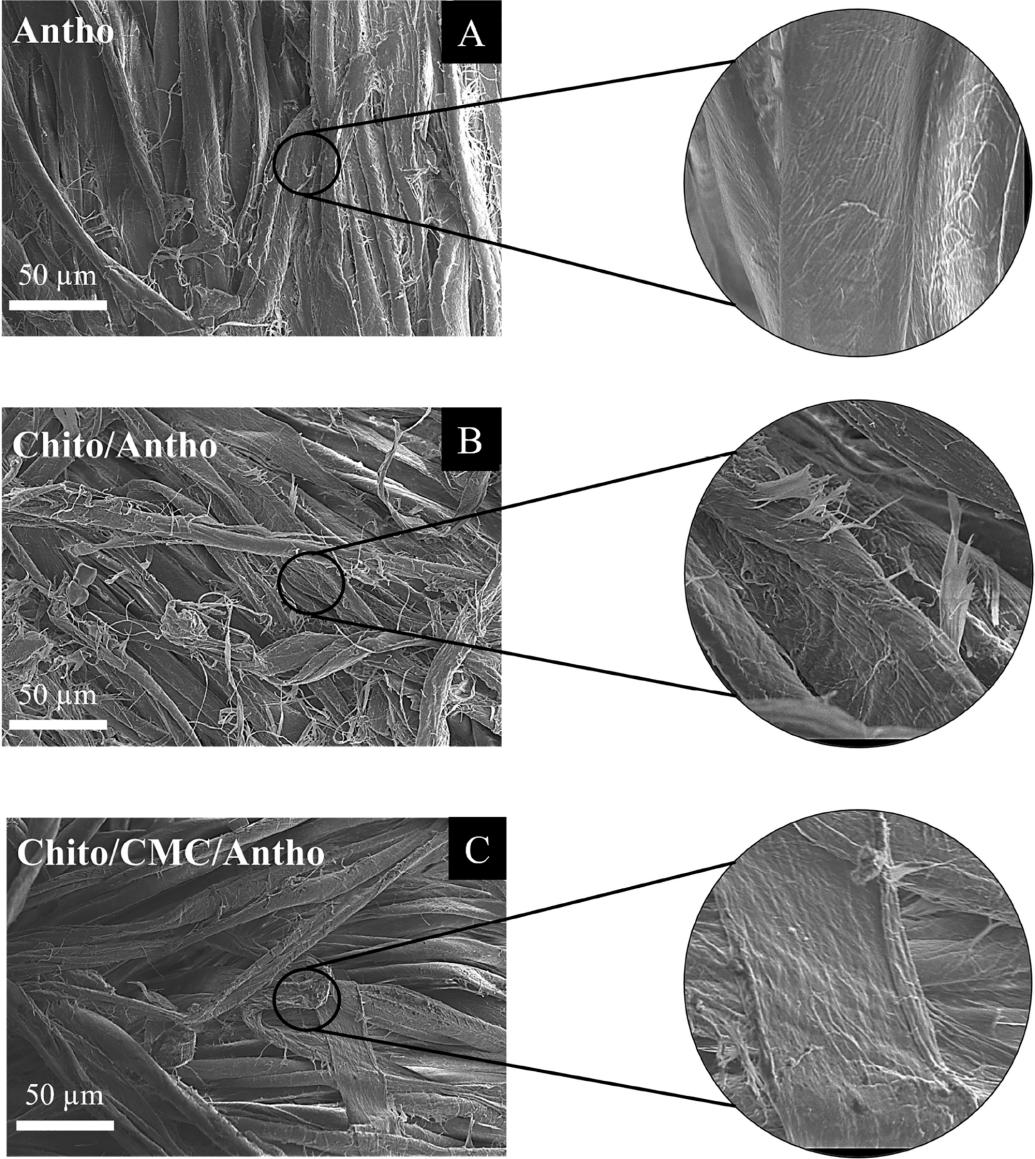
SEM images of cotton dyed with anthocyanin: (A) 500× without mordant; (B) 500× with chitosan as a mordant; and (C) 500× using chitosan/CMC as mordants.

The addition of CMC to the dyeing system results in an even more complex morphology (Figure 5C). However, the quantity of fibrils is lower compared to that of the sample with chitosan alone. These results suggest the formation of a more structured polymeric network, possibly due to the interaction between CMC and chitosan, which provides a more robust anthocyanin fixation. This structure has the potential to enhance the thermal stability and wear resistance, which could improve the durability of the dye.

Observations from SEM images highlight the effectiveness of chitosan/CMC agents in improving the fixation of anthocyanins on cotton fabrics. Chitosan, with its ability to form an adherent layer on the fibers, provides a robust base for anthocyanin retention. The addition of CMC seems to complement this structure, forming an even more stable and cohesive network that increases dye density and improves the fabric’s mechanical and durability properties. These results support the use of biopolymers as effective eco-friendly mordants, not only to improve the quality and durability of natural colors in fabrics but also to add desirable functionalities such as bacteriological resistance and higher stability to use and washing. Thus, the combination of chitosan and CMC represents a promising approach for the sustainable textile industry, offering a viable alternative to traditional synthetic mordants and dyes. Additionally, since these polymers are colorless, they do not alter the shade of the dye, which often occurs with the use of metallic mordants.

### 3.2 Impregnation of Lavender Oil in the Fabric

The impregnation of lavender oil onto a cotton fabric dyed with anthocyanin was achieved through two distinct methods. In the first method, the fabric was immersed in a solution of lavender oil in ethanol. In the second method, microcapsules based on CMC and chitosan containing lavender oil were produced. Due to the opposite charges of these two polymers, their mixing forms an insoluble complex. When this process is performed drop by drop under vigorous agitation, microcapsules are generated, encapsulating the oil within them. Encapsulation is primarily governed by polyelectrolyte complexation between cationic chitosan and anionic CMC, forming a stable polymeric network that traps oil droplets within the microcapsule structure. Although lavender oil is hydrophobic, it was initially emulsified using Tween 80, a nonionic surfactant, in an anthocyanin solution before being mixed with the chitosan phase. This emulsification process ensured the formation of finely dispersed oil droplets, preventing phase separation. Upon addition of the chitosan solution, the positively charged chitosan molecules interacted with the anionic groups of anthocyanin and the oil. When the CMC solution was introduced separately, it further facilitated capsule formation by promoting electrostatic interactions and hydrogen bonding between the polymers. This encapsulation method not only enhances the stability of the oil but also enables its controlled release, potentially extending the functional properties of the fabric. Anthocyanin was incorporated into the production of microcapsules to facilitate their observation under an optical microscope.

The determination of chitosan, CMC, and lavender oil concentrations was based on criteria previously established in the literature as well as on preliminary experiments aimed at optimizing microcapsule stability and encapsulation efficiency. The chitosan concentration of 0.5% (w/v) was selected based on studies on polyelectrolyte complexation systems,^20^ ensuring adequate viscosity for the formation of stable microcapsules and efficient electrostatic interactions with CMC. Higher concentrations resulted in excessive viscosity, hindering the homogeneous dispersion of the essential oil, whereas lower concentrations compromised capsule formation. The same concentration of CMC (DS 0.7–0.9)^42^ was chosen to balance the electrostatic interaction with chitosan, ensuring the formation of a cohesive polymeric network around the essential oil. The proportion of lavender oil was determined to maximize the encapsulation load without compromising the structural integrity of the microcapsules.^44^ The selection of these concentrations was validated through structural characterization of the capsules (optical microscopy and FTIR), as well as by assessing the essential oil release profile.^26^

The chemical interaction of the microcapsules with cotton fibers occurs primarily through electrostatic interactions and hydrogen bonding, as evidenced by the chemical properties of the biopolymers used. Chitosan, being cationic, and CMC, which exhibits anionic character in acidic media, strongly interact with the hydroxyl and carboxylate groups of the cotton fibers.^20^ These interactions promote the anchoring of the microcapsules to the fabric, creating a stable matrix that slows down the volatilization of lavender oil.^19,40^ The interaction between the molecules composing the microcapsules was assessed using FTIR, as shown in Figure 6. The key bands corresponding to each molecule are clearly visible. A detailed table listing each band and its corresponding functional group^52–54^ can be found in Table S1, and the FTIR spectra of chitosan, CMC and anthocyanin are in Figure S3 of SI.

**Figure 6:**
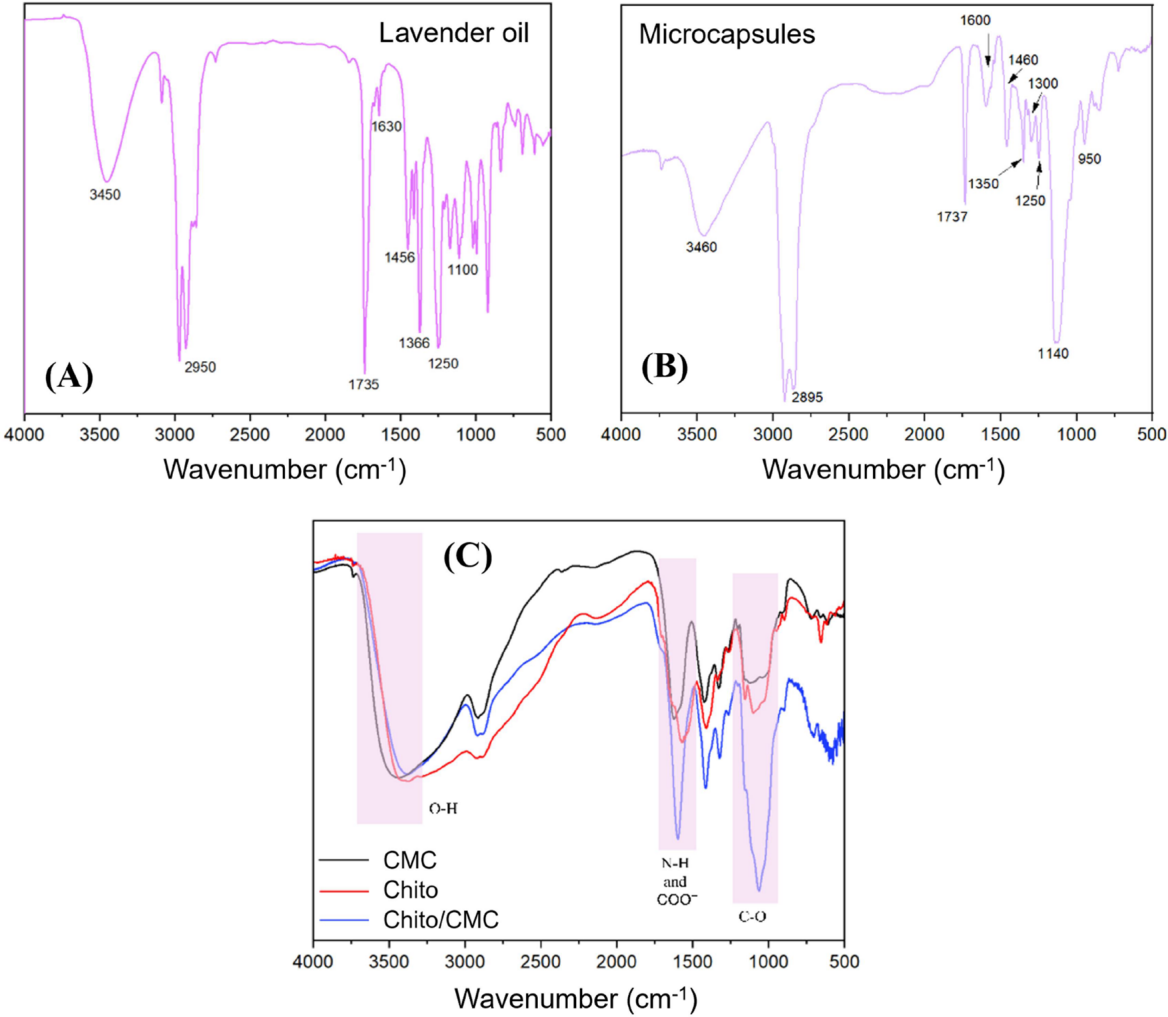
FTIR spectra of (A) a cast film containing lavender oil, (B) lavender microcapsules, and (C) CMC, Chito, and Chito/CMC.

The presence of lavender oil in the microcapsules was confirmed via FTIR analysis (Figure 6A). The spectrum highlights characteristic bands of linalool, linalyl acetate, and camphor, the main constituents of lavender oil, as previously described. Linalool presents a broad band at 3450 cm^–1^, corresponding to the O–H stretching of the hydroxyl group. Additionally, a strong band at 2950 cm^–1^ indicates the stretching of C–H bonds from methyl and methylene groups, typical of linalool’s aliphatic structure. The presence of a C=C double bond, characteristic of unsaturated compounds, is observed around 1630 cm^–1^, while the C–O stretching of the alcohol group appears near 1100 cm^–1^, confirming the presence of hydroxyl groups. In the case of linalyl acetate, an intense band around 1735 cm^–1^ corresponds to the stretching of the C=O bond in the ester group. Similar to linalool, the aliphatic groups of linalyl acetate show C–H stretching bands around 2950 cm^–1^ due to the presence of methyl and methylene groups. The band associated with the C–O–C stretching of the ester group appears around 1250 cm^–1^, while the C=C double bond, as seen in linalool, is observed near 1630 cm^–1^. Camphor, being a terpenic ketone, shows a C=O stretching band at around 1735 cm^–1^, which overlaps with the C=O band of linalyl acetate. Bands in the region of 2950 cm^–1^ reflect the C–H stretching of aliphatic groups in camphor, and the angular deformation of C–H bonds from these groups is observed at around 1366 cm^–1^. Thus, the FTIR spectrum of lavender oil clearly highlights the chemical functionalities of its main components, such as hydroxyl, ester, and ketone groups.^26,55,56^

The FTIR spectrum of the microcapsules (Figure 6B) shows a broad peak at approximately 3460 cm^–1^, attributable to the O–H and N–H stretching vibrations, indicating the abundant presence of hydroxyl and amine groups. These groups are significant structural components in chitosan, CMC, and anthocyanin. The peak around 2900 cm^–1^ is associated with C–H stretching vibrations, typical of the alkyl chains present in the polymeric structures of chitosan and CMC. In the region of 1600 cm^–1^, a characteristic peak of C=O (carbonyl) stretching vibrations is observed, which may be related to the amide I groups in chitosan and the carboxyl groups in CMC. The peak at approximately 1140 cm^–1^ can be attributed to C–O–C stretching vibrations, indicating the presence of ether linkages in the structures of chitosan and CMC. Finally, the peak at approximately 950 cm^–1^ is characteristic of out-of-plane C–H deformation vibrations in β-glycosidic rings, confirming the presence of glycosidic linkages in the polymeric structure. Additionally, the presence of a strong absorption band near 1740 cm^–1^ is a typical indication of ester functional groups, particularly in essential oils such as lavender oil, where linalyl acetate is a prominent compound. This ester band is often used as a marker in the identification and analysis of essential oils through FTIR spectroscopy.^55,56^ Tarhan et al.^26^ describes the band at 1740 cm^–1^ related to linalyl acetate and camphor as well as a broad OH band at 3660–3210 cm^–1^ attributed to OH stretching of linalool. Other characteristic bands of lavender oil are also described.

To study the interaction between CMC and chitosan in the complex formed during the preparation of the microcapsules, FTIR studies were also conducted (Figure 6C). Three regions of significant changes can be observed, as indicated in the spectrum of Figure 6. The shift of the band corresponding to the O–H stretching indicates the formation of hydrogen bonds between the hydroxyl groups (−OH) present in both chitosan and CMC. These hydrogen interactions can occur between the hydroxyls of chitosan and the carboxylate groups (COO−) of CMC, leading to a reorganization in the vibrational frequencies of the O–H group. The shift suggests a change in the chemical environment around these functional groups, reinforcing the hypothesis of direct interaction between the two macromolecules. The shift in the bands corresponding to the N–H group (from chitosan) and the carboxylate group (COO−) (from CMC) indicates an electrostatic interaction between the protonated amino groups of chitosan (NH_3_^+^) and the carboxylate groups of CMC. This type of interaction is common in polyelectrolyte complexes, where oppositely charged groups attract each other, stabilizing the formed complex.^57^

The synergistic interaction among chitosan, CMC, and anthocyanin significantly enhances the dye retention and stability. FTIR analysis reveals characteristic shifts in functional group signals, such as the broad peak at ∼3460 cm^–1^, indicating hydrogen bonding involving the hydroxyl and amino groups of chitosan, the carboxylate groups of CMC, and the hydroxyl groups of anthocyanins. Additionally, the peak at ∼1600 cm^–1^, associated with C=O stretching, suggests electrostatic interactions between the protonated amino groups of chitosan and the negatively charged carboxylate groups of CMC. Peaks around ∼1510 cm^–1^ related to aromatic C=C stretching may further indicate π-π stacking interactions involving anthocyanins.^9,18,44^ These findings are consistent with previous studies that highlight the ability of chitosan to act as a natural mordant, enhancing dye adsorption and stabilization through electrostatic and hydrogen bonding interactions. Based on these results and supported by the literature, these interactions collectively contribute to the enhanced retention and stabilization of anthocyanins within the polymer matrix. Electrostatic forces primarily anchor the anthocyanin molecules, while hydrogen bonding and π-π stacking provide additional stabilization, reducing molecular mobility and mitigating photodegradation pathways. These findings provide initial insights into the stabilizing role of this biopolymer system, laying the groundwork for further exploration of the long-term stability under varied conditions.

The increase in the intensity of these bands may also suggest a strengthening of intermolecular interactions within the complex. The C–O band can be related to both CMC and chitosan, and the increase and shift of this band can be attributed to the interaction between the two molecules, possibly through hydrogen bonding or other specific interactions involving the carboxylate group of CMC and the hydroxyl and amino groups of chitosan. This suggests structural reorganization of the molecules, possibly influencing the bonding between the polysaccharide chains and the C–O groups involved in the interactions. The shifts and increases in the bands observed in the FTIR spectrum indicate that the interactions between chitosan and CMC occur mainly through hydrogen bonds and electrostatic interactions between the functional groups of the two molecules. The formation of an insoluble complex can be explained by the interaction between the protonated amine groups (NH_3_^+^) of chitosan and the carboxylate groups (COO−) of CMC, which generate a denser, three-dimensional network that is less soluble in water.

Figure 7A displays the optical images of dry macroscopic capsules at 20× magnification, each with an approximate diameter of 1 mm. In Figure 7B, a capsule is depicted at 40× magnification prior to drying, revealing smaller microcapsules contained within the larger capsule. This image underscores the effectiveness of the encapsulation technique, showcasing a hierarchical structure in which numerous microcapsules are nested within the primary capsule. These internal microcapsules act as individual reservoirs for the oil, facilitating a controlled release mechanism. After 2 h of drying, as shown in Figure 7C, these microcapsules become indistinguishable, likely due to coalescence or shrinkage of the internal structure because of moisture or solvent loss. Figure 7D provides a more detailed view of the microcapsules within a capsule at 100× magnification, revealing diameters ranging from 10 to 80 μm. The histogram of the size distribution of the capsules is presented in Figure S4 of the Supporting Information, which shows that the average capsule diameter is 35 ± 11 μm. The histogram was generated by measuring the diameter of 100 individual capsules (Figure S5) using ImageJ software. The size variation among the microcapsules may impact the oil release profile, with smaller microcapsules potentially offering a faster release due to a higher surface-area-to-volume ratio, while larger microcapsules may ensure a more sustained release.

**Figure 7:**
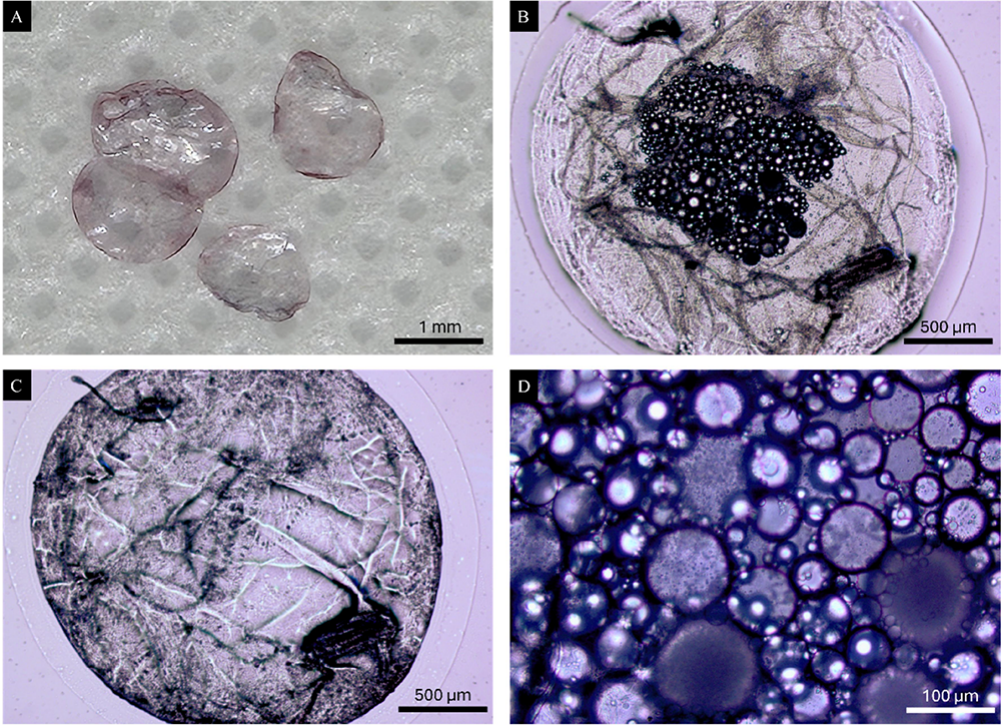
Macroscopic and microscopic images of microcapsules encapsulating lavender oil. (A) Dry macroscopic capsules at 20× magnification, showing an approximate diameter of 1 mm. Optical microscopy images of the (B) capsule at 40× magnification before drying, revealing numerous internal microcapsules, (C) Capsule after 2 h of drying at 40× magnification, where internal microcapsules become less distinguishable. (D) Microcapsules within a capsule at 100× magnification, displaying sizes ranging from 10 to 100 μm.

To evaluate the efficiency of encapsulation before incorporating the microcapsules into the fabric, a cast film was prepared on a quartz slide. The UV–vis absorbance spectrum was monitored over time to evaluate the decay of the absorption band at 310 nm (Figure 8A). Since lavender oil is soluble in ethanol, a comparative release study was also conducted in ethanol. In this analysis, three capsules were placed in a quartz cuvette containing ethanol, and an increase in absorbance was recorded over time (Figure 8B). To confirm that the observed absorption band corresponds to lavender oil, a reference spectrum of pure oil diluted in ethanol was obtained beforehand, identifying three primary absorption bands centered at 240, 280, and 310 nm.

**Figure 8:**
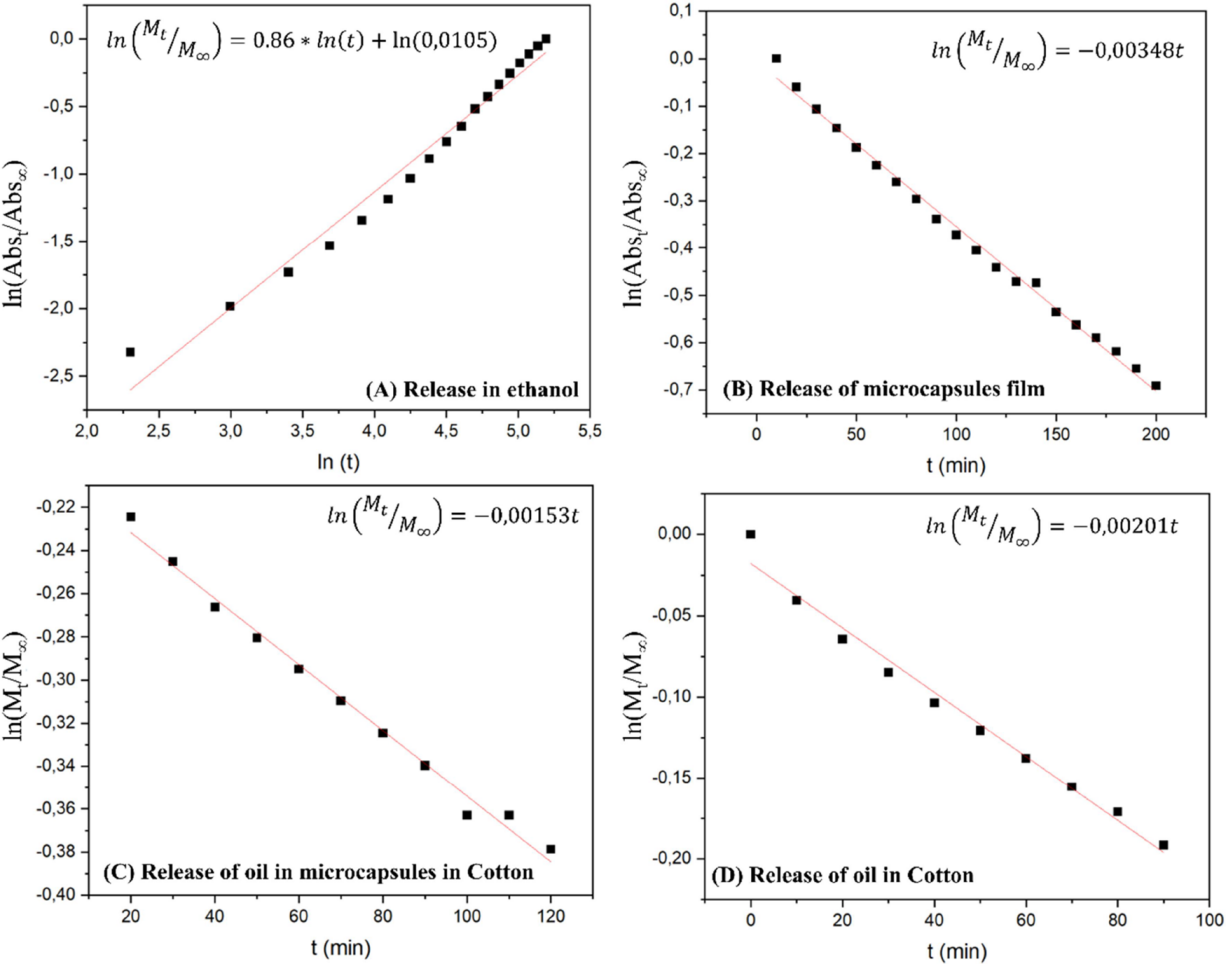
Analysis of lavender oil release (A) using microcapsules located in ethanol, (B) of a drop-cast film of microcapsules, (C) using microcapsules in cotton dyed, and (D) oil impregnated in dyed cotton.

The controlled release of lavender oil from chitosan-CMC microcapsules is driven by physical and chemical processes influenced by polymer properties, environmental conditions, and the encapsulated oil’s nature. When mixed, the oppositely charged chitosan and CMC interact electrostatically, forming a polyelectrolyte complex that encapsulates the oil within a stable gel network, ensuring a sustained release. During complex formation, the hydrophobic lavender oil is trapped within the polymeric network. The chitosan and CMC reorganize around the oil droplets, creating a microcapsule that protects the oil, thereby slowing its release.^16,58,59^

The Korsmeyer-Peppas model^59,60^ is extensively used in analyzing drug release kinetics from controlled release systems, such as polymeric matrices. This mathematical model is based on the relationship between the fraction of drug released (*M*_*t*_/*M*_*∞*_) and time (*t*), described by the equation $M_{t}/M_{\infty }=kt^{n}$ , where *k* is the diffusion constant and *n* is the exponent that indicates the predominant release mechanism. The value of (*n*) allows the identification of whether the release process is governed by Fickian diffusion, matrix relaxation, or an anomalous transport mechanism that combines both.^14,61^ This model is particularly useful in describing systems in which drug release is influenced by both diffusion and matrix erosion or swelling, making it especially applicable during the initial stages of release.

In addition to categorizing release profiles based on the value of the exponent (*n*), the Korsmeyer-Peppas model also enables a detailed interpretation of the transport mechanisms involved in the drug release process. When *n* ≤ 0.5, the mechanism is typically described as Fickian diffusion, where the oil moves passively through the polymeric matrix due to a concentration gradient, without significant alterations in the matrix structure. Conversely, for values of *n* between 0.5 and 1, the mass transport is classified as anomalous or non-Fickian, suggesting a combination of diffusion and polymer matrix relaxation, indicating that drug release may be influenced by both diffusion and the rearrangement of the matrix structure during the process.^62^ This model was employed to elucidate the release mechanism of oil encapsulated within microcapsules immersed in ethanol. Given that ethanol acts as a solvent with a high affinity for lavender oil, it facilitates efficient extraction of the oil into the surrounding medium. This swelling increases the permeability of the microcapsule walls, creating additional pathways for the oil to migrate outward. Consequently, the combination of solvent affinity and structural changes in the microcapsules under ethanol exposure plays a crucial role in the efficient release and diffusion of the encapsulated oil into the external environment.

In Figure 8A, the graph of ln­(Abs_*t*_/Abs_∞_) versus ln­(*t*) illustrates the release kinetics of the oil in ethanol. From this analysis, we determined a diffusion coefficient of 1.05 × 10^–2^ min^–1^. This value further supports the model’s prediction that ethanol, due to its solvent properties and its ability to induce swelling in the microcapsules, significantly enhances the diffusion process compared with the release in the air. The linearity of the graph suggests a consistent release mechanism, confirming the role of ethanol not only in facilitating the release but also in controlling the rate of diffusion of the encapsulated oil into the surrounding medium.

When the release occurs in a gaseous medium, such as the release into the atmosphere, the process is influenced neither by the swelling of the polymeric matrix, which typically facilitates diffusion, nor by the solvent’s affinity with the oil, which could aid in the transport process. In this scenario, diffusion is primarily driven by the volatility of the oil. The most appropriate model for studying this type of release is first-order kinetics.^63^ In this model, the rate of oil diffusion is directly proportional to the remaining concentration of the oil. This implies that as the oil evaporates and its concentration decreases, the evaporation rate also diminishes exponentially, leading to a decay curve characteristic of first-order processes.

Furthermore, the analysis of the plots of ln­(Abs_*t*_/Abs_∞_) or ln­(*M*_t_/*M*_∞_) versus *t* for these systems yields a straight line, where the slope is equal to *–k*, the rate constant. This linear relationship underscores the suitability of the first-order kinetic model in describing the release of volatile compounds into the atmosphere.

The curves depicted in Figure 8B–D represent the release profiles of lavender oil through a cast film of microcapsules, microcapsules impregnated into dyed cotton fabric, and lavender oil directly impregnated into the dyed cotton fabric, respectively. The diffusion constants determined for these studies were 3.48 × 10^–3^ min^–1^ for the microcapsule film, 1.53 × 10^–3^ min^–1^ for the microcapsule-impregnated fabric containing lavender oil, and 2.01 × 10^–3^ min^–1^ for the fabric impregnated with lavender oil. It is noteworthy that the microcapsule-impregnated fabric containing lavender oil exhibited the lowest diffusion constant, indicating a slower diffusion rate, which was expected. The presence of microcapsules, along with the structure of the fabric and the layers involved in the dyeing process, creates additional barriers to volatilization. This slower diffusion is attributed to the enhanced interactions between the oil molecules and various components of the release matrix, including the polymeric microcapsules and the fibers of the fabric. These interactions likely hinder the free movement of the oil, thereby reducing its evaporation rate. These results highlight the effectiveness of using microcapsules in conjunction with fabric matrices to control the release of volatile compounds. The slower release rate can be particularly advantageous in applications in which prolonged fragrance retention or extended release of active ingredients is desired. Moreover, the study underscores the importance of considering the physical and chemical interactions within the release matrix as they significantly influence the diffusion kinetics and overall performance of the delivery system.

## 4 Conclusions

This study demonstrates the potential of chitosan- and CMC-based microcapsules for controlled lavender oil release in anthocyanin-dyed cotton fabrics. The combination enhances fragrance longevity, dye stability, and functional properties, supporting eco-friendly textile innovations. The anthocyanin dyeing process, enhanced using chitosan/CMC as mordants, not only provided vibrant and stable coloration but also augmented the fabric’s aesthetic and functional characteristics. Our results demonstrated that the use of microcapsules impregnated into dyed cotton fabric significantly slows the release of lavender oil compared with both the microcapsule film and the direct impregnation of oil into the fabric. The lower diffusion constant observed for the microcapsule-impregnated fabric indicates a more controlled and sustained release, which is advantageous for applications requiring prolonged fragrance or active ingredient delivery. This retardation in release can be attributed to the complex interactions among the oil, the polymeric microcapsules, and the fabric matrix, which creates additional barriers to volatilization. The findings highlight the potential of utilizing microcapsule technology in textile applications to achieve tailored release profiles, offering new avenues for enhancing the functionality of treated fabrics in various industries. Future studies exploring variations in the chitosan-to-CMC ratio and other encapsulation parameters will further optimize the release profiles for tailored applications, enhancing the versatility of this approach. Besides, this study contributes to the growing field of smart textiles, offering innovative solutions for sustainable and effective fragrance delivery while addressing environmental concerns in various industrial and consumer applications from therapeutic garments to high-performance sportswear.

## Supplementary Material



## Notes

UV–vis spectrum of anthocyanin solution; fabric images with different mordants; FTIR spectra of chitosan, CMC, and anthocyanin; capsule size distribution histogram; and individual microcaps (PDF)

## References

[1] Hossain M. T., Shahid M. A., Limon M. G. M., Hossain I., Mahmud N. (2024). Techniques,
applications, and challenges in textiles for a sustainable future. Journal of Open Innovation: Technology, Market, and Complexity..

[2] Ardila-Leal L. D., Poutou-Piñales R. A., Pedroza-Rodríguez A. M., Quevedo-Hidalgo B. E. (2021). A Brief History of Colour, the Environmental Impact
of Synthetic Dyes and Removal by Using Laccases. Molecules..

[3] Pizzicato B., Pacifico S., Cayuela D., Mijas G., Riba-Moliner M. (2023). Advancements
in Sustainable Natural Dyes for Textile Applications: A Review. Molecules..

[4] Alappat B., Alappat J. (2020). Anthocyanin pigments: Beyond aesthetics. Molecules..

[5] Khoo H. E., Azlan A., Tang S. T., Lim S. M. (2017). Anthocyanidins and
anthocyanins: Colored pigments as food, pharmaceutical ingredients,
and the potential health benefits. *Food*. Nutr. Res. (N.Y.).

[6] Li Q., Zhang F., Wang Z., Feng Y., Han Y. (2023). Advances in
the Preparation, Stability, Metabolism, and Physiological Roles of
Anthocyanins: A Review. Foods..

[7] Kamiloglu S., Van Camp J., Capanoglu E. (2018). Black carrot
polyphenols: effect
of processing, storage and digestion—an overview. Phytochemistry Reviews..

[8] Nassour, R. ; Ayash, A. ; Al-Tameemi, K. Anthocyanin pigments: Structure and biological importance. www.jchps.com.

[9] Grande R., Räisänen R., Dou J. (2023). In Situ
Adsorption of Red Onion (Allium cepa) Natural Dye on Cellulose Model
Films and Fabrics Exploiting Chitosan as a Natural Mordant. ACS Omega..

[10] Radhakrishnan A., Panicker U. G. (2025). Sustainable chitosan-based
biomaterials for the future:
a review. Polym. Bull..

[11] Leceta I., Guerrero P., Cabezudo S., de la Caba K. (2013). Environmental
assessment of chitosan-based films. J. Clean.
Prod..

[12] Priyadarshi R., Rhim J. W. (2020). Chitosan-based biodegradable
functional films for food
packaging applications. Innovative Food Science
& Emerging Technologies..

[13] Desai N., Rana D., Salave S. (2023). Chitosan:
A Potential
Biopolymer in Drug Delivery and Biomedical Applications. Pharmaceutics..

[14] Matussek F., Pavinatto A., Knospe P., Beuermann S., Sanfelice R. C. (2022). Controlled Release of Tea Tree Oil from a Chitosan
Matrix Containing Gold Nanoparticles. Polymers.

[15] Pacheco K. M. L., Torres B. B. M., Sanfelice R. C. (2023). Chitosan and chitosan/turmeric-based
membranes for wound healing: Production, characterization and application. Int. J. Biol. Macromol..

[16] da
Silva B. B., Menezes J. E., da Costa M. M., dos Santos L., Sanfelice R. C., Pavinatto A. (2024). Comparison of the acyclovir release
carried in chitosan-based microstructured systems. Polymer Bulletin..

[17] Pirsa S., Hafezi K. (2023). Hydrocolloids: Structure,
preparation method, and application
in food industry. Food Chem..

[18] Aguado R. J., Mazega A., Tarrés Q., Delgado-Aguilar M. (2023). The role of
electrostatic interactions of anionic and cationic cellulose derivatives
for industrial applications: A critical review. Ind. Crops Prod..

[19] Rahman M. S., Hasan M. S., Nitai A. S. (2021). Recent
developments
of carboxymethyl cellulose. Polymers.

[20] Zhang S., Liu W., Liang J. (2013). Buildup mechanism of carboxymethyl cellulose
and chitosan self-assembled films. Cellulose..

[21] Cheng Y., Hu Z., Zhao Y. (2019). Sponges of carboxymethyl chitosan grafted with
collagen peptides for wound healing. Int. J.
Mol. Sci..

[22] Chen B. H., Inbaraj B. S. (2019). Nanoemulsion and nanoliposome based strategies for
improving anthocyanin stability and bioavailability. Nutrients.

[23] Soliman E. A., El-Moghazy A. Y., El-Din M. S. M., Massoud M. A. (2013). Microencapsulation
of Essential Oils within Alginate: Formulation and &amp;lt;i&amp;gt;in
Vitro&amp;lt;/i&amp;gt; Evaluation of Antifungal Activity. J. Encapsulat. Adsorpt. Sci..

[24] Marudova M., MacDougall A. J., Ring S. G. (2004). Pectin–chitosan interactions
and gel formation. Carbohydr. Res..

[25] De
Groot A., Schmidt E. (2016). Essential Oils, Part V: Peppermint
Oil, Lavender Oil, and Lemongrass Oil. Dermatitis..

[26] Tarhan İ, Çelikten Ş, Kestek H. M. (2023). Development
of a new and rapid FTIR method using chemometric modeling techniques
for the determination of lavandin adulteration in lavender essential
oil. Vib Spectrosc..

[27] Vareltzis P., Fotiou D., Papatheologou V., Kyroglou S., Tsachouridou E., Goula A. M. (2024). Optimized Solid–Liquid
Separation of Phenolics
from Lavender Waste and Properties of the Dried Extracts. Separations..

[28] Poutaraud A., Guilloteau L., Gros C. (2018). Lavender
essential oil
decreases stress response of horses. Environ.
Chem. Lett..

[29] Tomi K., Kitao M., Murakami H., Matsumura Y., Hayashi T. (2018). Classification of lavender essential oils: sedative
effects of Lavandula oils. Journal of Essential
Oil Research..

[30] Karaca N., Demirci B., Gavahian M., Demirci F. (2023). Enhanced Bioactivity
of Rosemary, Sage, Lavender, and Chamomile Essential Oils by Fractionation,
Combination, and Emulsification. ACS Omega..

[31] Manzoor A., Asif M., Khalid S. H., Ullah Khan I., Asghar S. (2023). Nanosizing of Lavender, Basil, and Clove Essential
Oils into Microemulsions for Enhanced Antioxidant Potential and Antibacterial
and Antibiofilm Activities. ACS Omega..

[32] Shady K., Nair J. M. C., Crannell C. (2019). Lavender aromatherapy: Examining
the effects of lavender oil patches on patients in the hematology-oncology
setting. Clin J. Oncol Nurs..

[33] Cavanagh H. M. A., Wilkinson J. M. (2002). Biological
activities of lavender essential oil. Phytotherapy
Research..

[34] Massella D., Giraud S., Guan J., Ferri A., Salaün F. (2019). Textiles for
health: a review of textile fabrics treated with chitosan microcapsules. Environ. Chem. Lett..

[35] Ömeroğulları
Başyiğit Z., Kut D., Yenilmez E., Eyüpoğlu Ş, Hocaoğlu E., Yazan Y. (2018). Vitamin E loaded fabrics as cosmetotextile products: Formulation
and characterization. Text. Apparel..

[36] Valle J. A.
B., Rita de Valle C. S. C., Bierhalz ACK, Bezerra F. M., Hernandez A. L., Lis Arias M. J. (2021). Chitosan microcapsules: Methods of
the production and use in the textile finishing. J. Appl. Polym. Sci..

[37] Teixeira C.
S. N. R., Martins I. M. D., Mata V. L. G., Filipe
Barreiro M. F., Rodrigues A. E. (2012). Characterization and evaluation of
commercial fragrance microcapsules for textile application. J. Text. Inst..

[38] Eyupoglu S., Kut D., Girisgin A. O. (2019). Investigation
of the bee-repellent properties
of cotton fabrics treated with microencapsulated essential oils. Textile Research Journal..

[39] Baranwal J., Barse B., Fais A., Delogu G. L., Kumar A. (2022). Biopolymer:
A Sustainable Material for Food and Medical Applications. Polymers (Basel)..

[40] Özsevinç A., Alkan C. (2023). Polyurethane shell medicinal lavender release microcapsules for textile
materials: An environmentally friendly preparation. Ind. Crops Prod..

[41] Özsevinç A., Alkan C. (2022). Ethylene glycol
based polyurethane shell microcapsules for textile
applications releasing medicinal lavender and responding to mechanical
stimuli. Colloids Surf., A.

[42] Ge J., Yue P., Chi J., Liang J., Gao X. (2018). Formation and stability
of anthocyanins-loaded nanocomplexes prepared with chitosan hydrochloride
and carboxymethyl chitosan. Food Hydrocoll..

[43] Javanmardi Z., Koushesh Saba M., Amini J., Nourbakhsh H. (2022). Application
of Nanoemulsions of Thyme and Peppermint Essential Oils with Carboxymethyl
Cellulose Coating on Postharvest Longevity of Strawberry. Iran. J. Biosyst. Eng..

[44] Khanna S., Sharma S., Chakraborty J. N. (2015). Performance
assessment of fragrance
finished cotton with cyclodextrin assisted anchoring hosts. Fashion Text..

[45] Askar K. A., Alsawad Z. H., Khalaf M. N. (2015). Evaluation of the
pH and thermal
stabilities of rosella anthocyanin extracts under solar light. Beni Suef Univ J. Basic Appl. Sci..

[46] Jackman R. L., Yada R. Y., Tung M. A., Speers R. A. (1987). Anthocyabubs as
food colorants: A Review. J. Food Biochem..

[47] Kou S., Peters L., Mucalo M. (2022). Chitosan: A review of molecular structure,
bioactivities and interactions with the human body and micro-organisms. Carbohydr. Polym..

[48] Osorio
Echavarría J., Gómez Vanegas N. A., Orozco C. P. O. (2022). Chitosan/carboxymethyl
cellulose wound dressings supplemented with biologically synthesized
silver nanoparticles from the ligninolytic fungus Anamorphous Bjerkandera
sp. R1. Heliyon..

[49] Macedo J. B., Sanfelice R. C., Mercante L. A. (2022). Antimicrobial Activity
of Chitosan and Its Derivatives: Influence of Its Structural Characteris. Quim Nova..

[50] Liu Y., Tikunov Y., Schouten R. E., Marcelis L. F. M., Visser R. G. F., Bovy A. (2018). Anthocyanin Biosynthesis and Degradation Mechanisms
in Solanaceous Vegetables: A Review. Front Chem..

[51] Kuswandi B., Asih N. P. N., Pratoko D. K., Kristiningrum N., Moradi M. (2020). Edible pH sensor based on immobilized
red cabbage anthocyanins
into bacterial cellulose membrane for intelligent food packaging. Packaging Technology and Science..

[52] Lima, G. ; Gabrieli De Souza, A. NANOCELULOSE COMO REFORÇO EM HIDROGÉIS SUPERABSORVENTES DE CARBOXIMETILCELULOSE. 2020. https://www.researchgate.net/publication/339593309.

[53] Bhushan B., Bibwe B., Pal A. (2023). FTIR spectra, antioxidant
capacity and degradation kinetics of maize anthocyanin extract under
variable process conditions: Anthocyanin degradation under storage. Appl. Food Res..

[54] Queiroz M. F., Melo K. R. T., Sabry D. A., Sassaki G. L., Rocha H. A. O. (2015). Does
the use of chitosan contribute to oxalate kidney stone formation?. Mar. Drugs.

[55] Samfira I., Rodino S., Cristina R. T. (2015). Characterization and
Identity Confirmation of Essential Oils by Mid Infrared Absorption
Spectrophotometry. Dig. J. Nanomater. Biostruct..

[56] Agatonovic-Kustrin S., Ristivojevic P., Gegechkori V., Litvinova T. M., Morton D. W. (2020). Essential Oil Quality
and Purity Evaluation via FT-IR
Spectroscopy and Pattern Recognition Techniques. Appl. Sci..

[57] Lankalapalli S., Kolapalli V. R. M. (2009). Polyelectrolyte
complexes: A review of their applicability
in drug delivery technology. Indian J. Pharm.
Sci..

[58] Pascoal K. L. L., Siqueira S. M. C., de
Amorim A. F. V. (2021). Physical-chemical characterization, controlled
release,
and toxicological potential of galactomannan-bixin microparticles. J. Mol. Struct..

[59] Dash S., Murthy P. N., Nath L., Chowdhury P. (2010). Kinetic modeling
on drug release from controlled drug delivery systems. Acta Pol. Pharm..

[60] Schneider R., Mercante L. A., Andre R. S., de Brandão H. M., Mattoso L. H. C., Correa D. S. (2018). Biocompatible electrospun nanofibers
containing cloxacillin: Antibacterial activity and effect of pH on
the release profile. React. Funct. Polym..

[61] Fu Y., Kao W. J. (2010). Drug release kinetics
and transport mechanisms of non-degradable
and degradable polymeric delivery systems. Expert
Opin Drug Delivery.

[62] Altan A., Aytac Z., Uyar T. (2018). Carvacrol loaded electrospun
fibrous
films from zein and poly­(lactic acid) for active food packaging. Food Hydrocoll..

[63] Ansar R., Saqib S., Mukhtar A. (2022). Challenges
and recent
trends with the development of hydrogel fiber for biomedical applications. Chemosphere.

