# Supplementary material for: Anthocyanin-Dyed Cotton Enhanced with Lavender Oil Microcapsules: A Dual Approach for Color Stability and Sustained Fragrance Release
Source: ACS Omega. 2025 May 30;10(22):22459–71. doi: 10.1021/acsomega.4c09486 (PMC12163680; doi:10.1021/acsomega.4c09486)
Supplement: Supplementary file 1 [file ao4c09486_si_001.pdf]

Supporting Information

Anthocyanin-Dyed Cotton Enhanced with Lavender Oil Microcapsules: A Dual Approach for Color Stability and Sustained Fragrance Release

Rafael Grande[1], Kelcilene B. R. Teodoro[2], Isabela S. Bertho[3], Isabela F. Pinheiro[3], Alessandra R. P. Ambrozin[3], Daniel S. Correa[2], Débora T. Balogh[4], Rafaela C. Sanfelice[3,4*]

[1] Grande Apresentações, Rua Paraná, 229, Centro, Poços de Caldas, Minas Gerais, 37701-018, Brazil.

[2] Nanotechnology National Laboratory for Agriculture (LNNA), Embrapa Instrumentation, Rua XV de Novembro, 1452, Centro, São Carlos, São Paulo, 13561-206, Brazil.

[3] Science and Technology Institute, Federal University of Alfenas (ICT/UNIFAL), Rodovia José Aurélio Vilela, 11999, BR 267 Km 533, Poços de Caldas, Minas Gerais, 37715-400, Brazil.

[4] São Carlos Institute of Physics, University of São Paulo, Av. Trabalhador São-Carlense, 400, Parque Arnold Schimidt, São Carlos, SP, 13566-590, Brazil.

*Corresponding author: rafaela.sanfelice@unifal-mg.edu.br

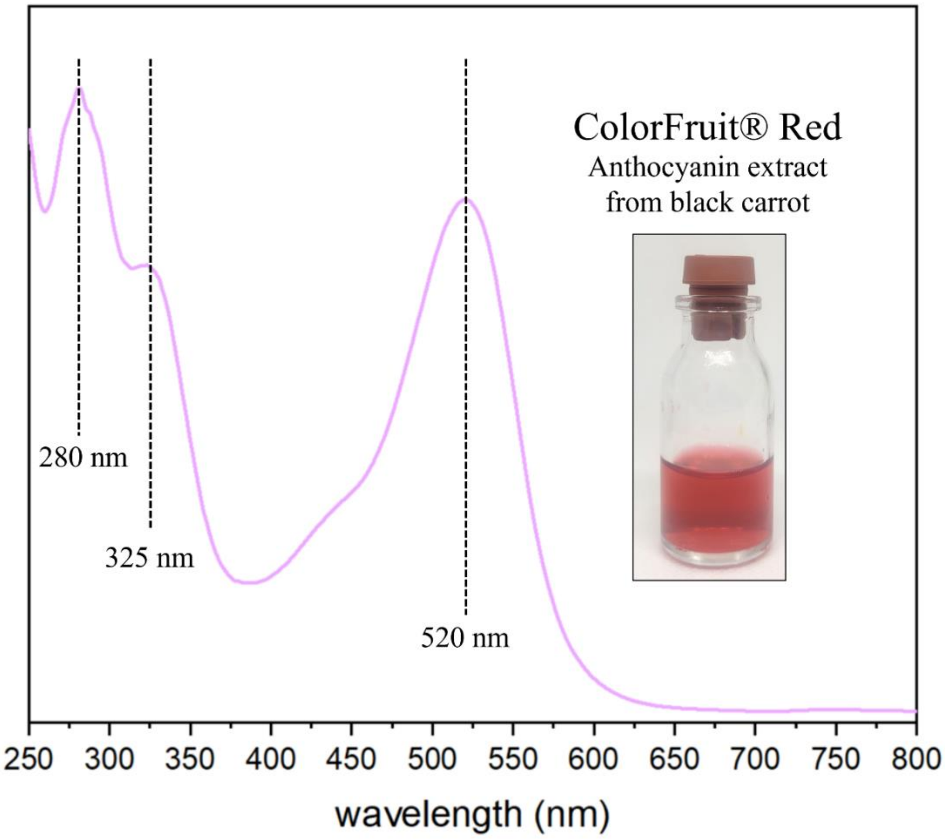

Figure S1: UV-Vis spectrum of commercial anthocyanin extract from black carrot (ColorFruit® Red) solution and the inset shows a digital picture of the solution

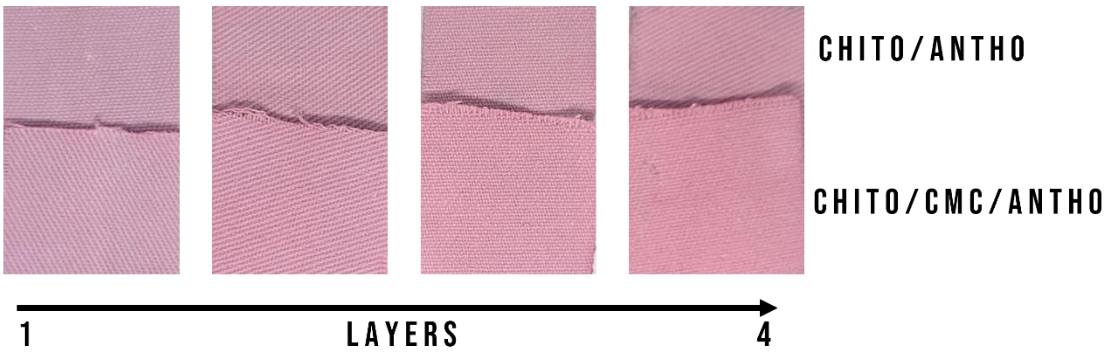

Figure S2: Image of fabrics dyed with anthocyanin using chitosan and chitosan/CMC as mordants with 1, 2, 3, and 4 layers.

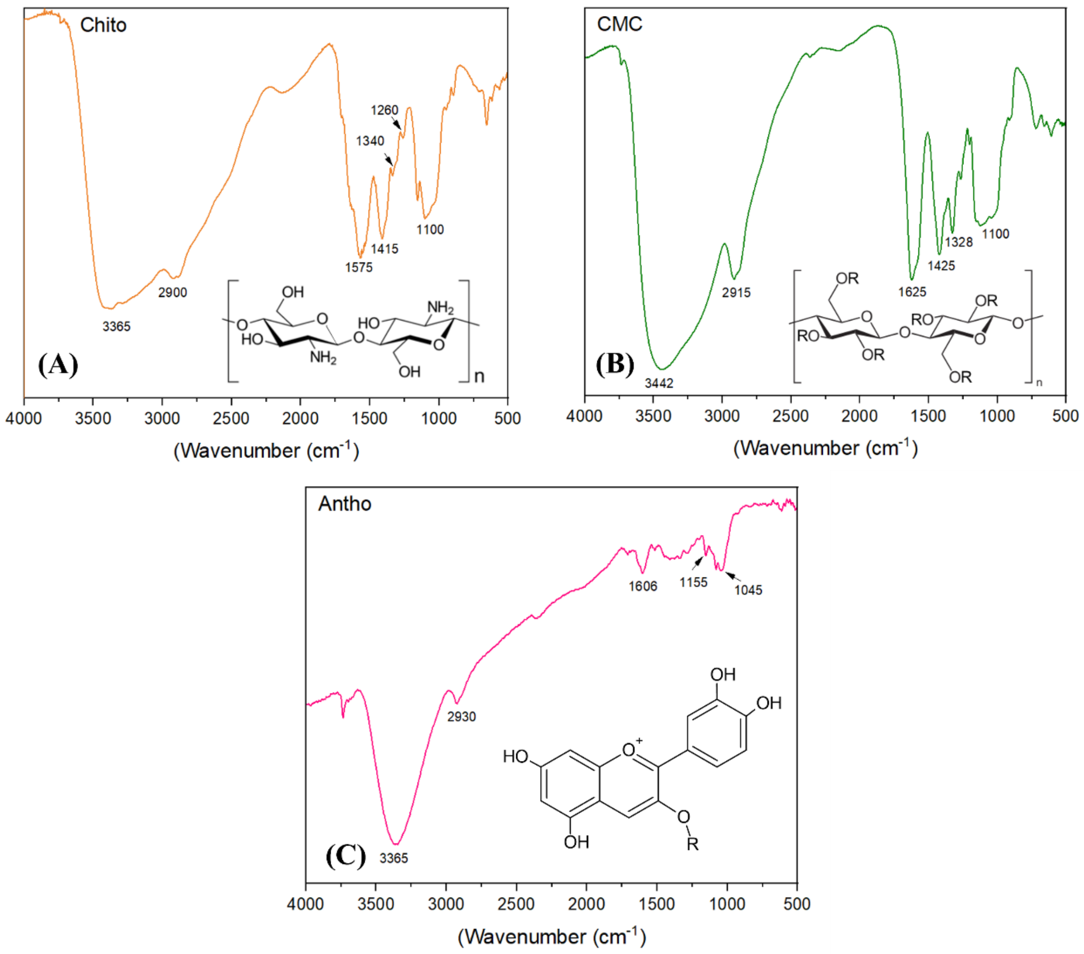

Figure S3: FTIR Spectrum of a Cast Film Containing (A) Chitosan, (B) CMC, and (C) Anthocyanin

Table S1: Main FTIR bands and their corresponding functional groups in chitosan, CMC, and anthocyanin.

	cm^{-1}	Functional Group
	3365	Stretching vibration of hydroxyl (–OH) and amine (–NH) groups
	2900	The stretching of aliphatic C–H bonds.
	1575	The bending vibration of the amine group (N–H).
Chito	1415	$–CH_3$, $–CH_2–$ or –OH (glucosamine ring) groups
	1340	C–N stretching vibration.
	1260	C–O–C stretching
	1160	C–O–C stretching
	1100	C–O stretching

CMC	3442	The stretching of hydroxyl (–OH)
	2915	The stretching of aliphatic C–H bonds.
	1625	Asymmetric stretching of the carboxylate group (–COO⁻).
	1425	Bending of the CH_2 group
	1328	C–H bending vibration and C–O stretching.
	1100	C–O stretching
Antho	3365	The stretching of hydroxyl (–OH)
	2930	The stretching of aliphatic C–H bonds.
	1606	Aromatic ring stretching (C=C).
	1155	C–O stretching of ethers.
	1045	C–O stretching

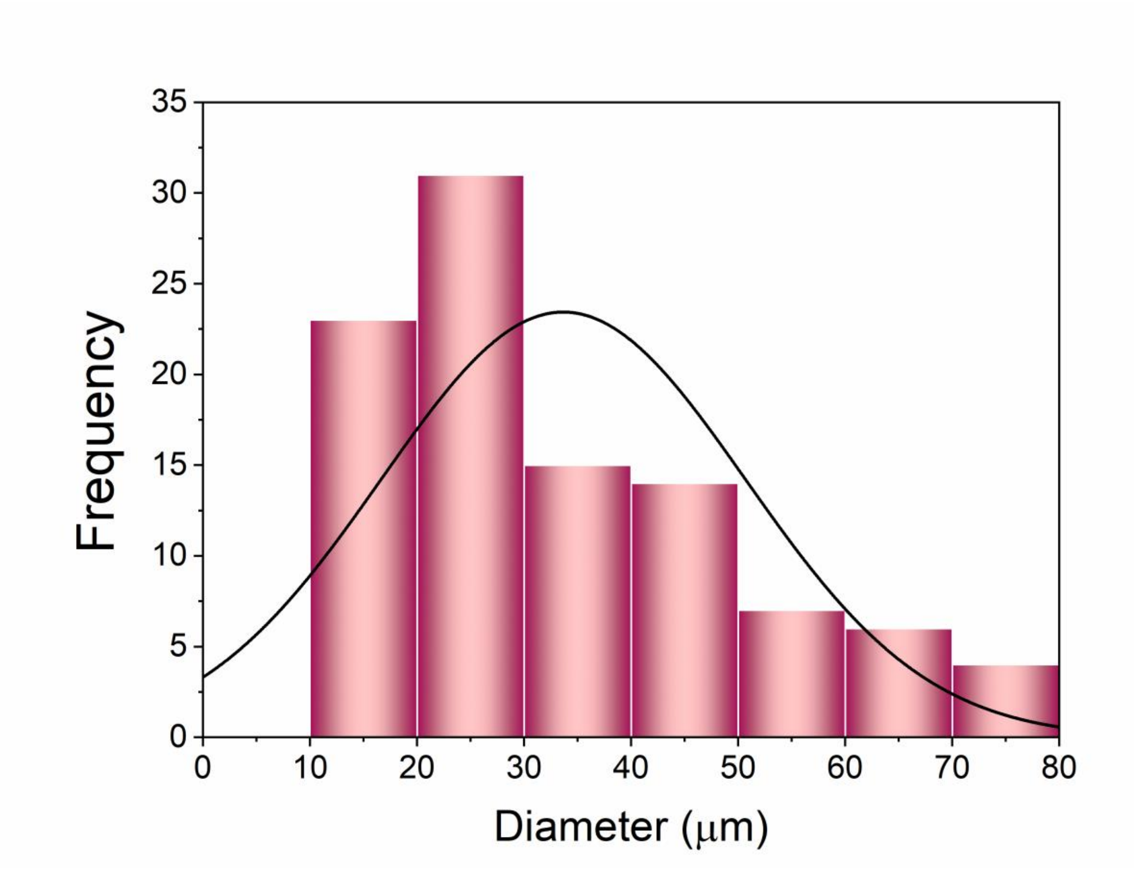

Figure S4: Histogram of the size distribution of chitosan and CMC microcapsules containing lavender oil.

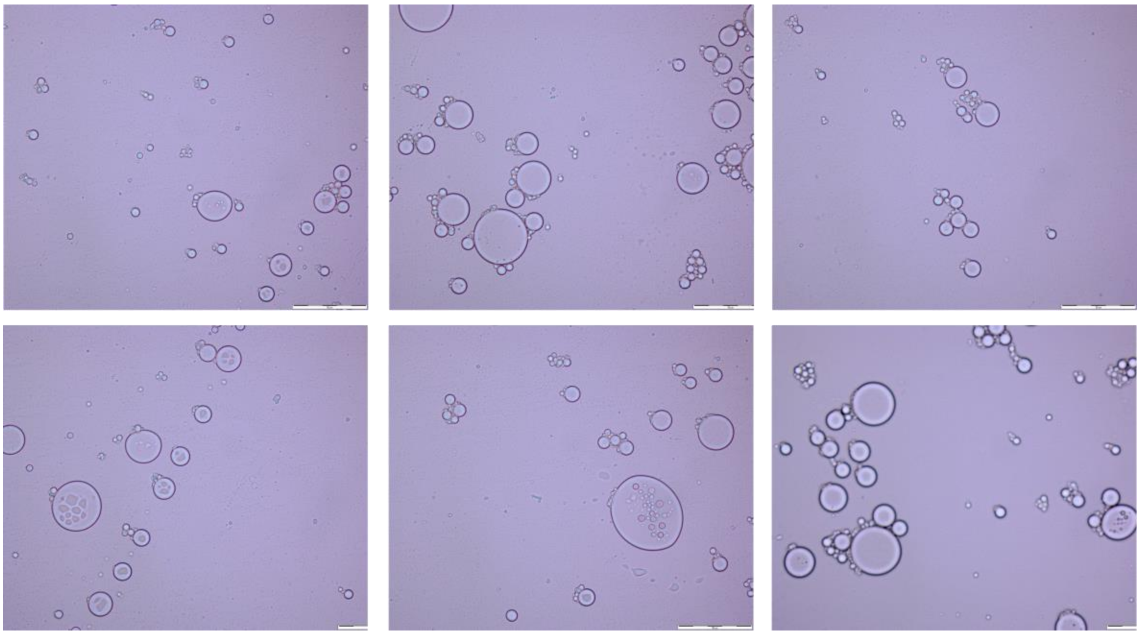

Figure S5: Microscopy images of microcapsules used for size distribution analysis. These images were processed using ImageJ software to measure the diameters of one hundred (100) individual capsules, generating the histogram presented in Figure S4.
